# Coronary artery calcium score and coronary computed tomography angiography predict one-year mortality in patients with type 2 diabetes and peripheral artery disease undergoing partial foot amputation

**DOI:** 10.1177/14791641221125190

**Published:** 2022-10-12

**Authors:** Evgeniya Shalaeva, Arjola Bano, Ulugbek Kasimov, Bakhtiyor Janabaev, Iris Baumgartner, Markus Laimer, Hugo Saner

**Affiliations:** 1 Graduate School for Health Sciences, 27210 University of Bern, Bern, Switzerland; 2 187926 Tashkent Medical Academy, Tashkent, Uzbekistan; 3 Institute for Social and Preventive Medicine, 27210 University of Bern, Bern, Switzerland; 4 Department of Cardiology, Inselspital, Bern University Hospital, 27210 University of Bern, Bern, Switzerland; 5 Department of Clinical and Interventional Angiology, University Hospital Bern, 549043 Swiss Cardiovascular Centre, Bern, Switzerland; 6 Clinic for Diabetology, Endocrinology, Nutrition and Metabolism, University Hospital Bern, Bern Switzerland

**Keywords:** Coronary artery disease, coronary computed tomographic angiography, coronary artery calcium score, type 2 diabetes, peripheral artery disease, partial foot amputation

## Abstract

The aim of the study was to examine the predictive value of coronary computed tomographic angiography (CCTA) and coronary artery calcium score (CACS) on 1-year all-cause mortality in type 2 diabetes (T2D) patients with peripheral artery disease (PAD) undergoing partial foot amputations (PFA).

**Methods:**

This is a single-center prospective cohort study including 199 consecutive patients with T2D, PAD (mean age 62.3 ± 7.2 years; 62.8% males), and preoperative CACS and CCTA undergoing PFA and followed-up over 1 year.

**Results:**

Over a period of 1 year follow-up, a total of 35 (17.6%) participants died. The area under ROC curve to predict mortality for the CACS was 0.835 (95% CI:0.769–0.900), for CCTA 0.858 (95% CI:0.788–0.927). After adjustment for confounders, compared to no-stenosis on CCTA (reference), the risk of all-cause mortality in non-obstructive coronary atery disease (CAD) increased (HR = 1.38, 95% CI [0.75–12.86], *p* = .284), 1-vessel obstructive CAD (HR = 8.13, 95% CI [0.87–75.88], *p* = .066), 2-vessels (HR = 10.94, 95% CI [1.03–115.8], *p* = .047), and 3-vessels (HR = 45.73, 95% CI [4.6–454.7], *p* = .001) respectively. Increasing levels of CACS tended to be associated with increased risk of all-cause mortality (HR = 1.002, 95% CI [1.0–1.003], *p* = .061). 61/95 patients with obstructive CAD underwent coronary revascularization.

**Conclusions:**

Coronary artery calcium score and CCTA have a high predictive value for 1-year all-cause mortality in T2D patients undergoing minor amputations and may be considered for preoperative risk assessment allowing timely preventive interventions.

## Introduction

Partial foot amputation (PFA) in patients with type 2 diabetes (T2D) and peripheral artery disease (PAD) carries a high risk for cardiovascular complications,^1,2^ which might often be underestimated by considering as a low risk surgery.^3,4^ Partial foot amputation is often semi-urgent because of spreading infection with septicemia. These circumstances may be encountered more often in countries with lower socioeconomic status where patients are less likely to seek medical help and possibly present later with more advanced disease stages. Under these circumstances, coronary angiography may not be readily available and less costly alternative procedures are used for preoperative risk assessment. Exercise ECG testing, stress myocardial perfusion scintigraphy, or stress echocardiography for the detection of obstructive coronary artery disease (CAD) are often contraindicated in these patients because of lower limb lesions, foot infection, requiring emergency foot surgery.^5,6,7^

Over 50% of T2D patients die as a consequence of CAD.^8^ Patients with PAD have a particularly high mortality rate from major adverse cardiovascular events (MACE).^9,4^ However, atherosclerotic cardiovascular diseases as CAD are largely underdiagnosed and undertreated in patients undergoing minor limb amputations.^10^ It is well known that obstructive CAD has been observed in 54%–69% of patients with critical PAD.^11,12^ However, the effectiveness of preoperative evaluation of coronary arteries in patients with non-critical PAD is unclear. Revised cardiac risk index (RCRI) was associated with both 30-days and 1-year mortality in patients undergoing lower extremity vascular surgery.^13^ Taking into account that 3-years mortality after below-knee amputations in T2D patients is estimated to be close to 40–60%,^14^ with most deaths attributed to CAD, predictive value of non-invasive coronary computed tomographic angiography (CCTA) and coronary artery calcium score (CACS) may allow timely preventive measures to improve long-term outcomes.

The aim of the study was to observe the severity of coronary obstruction in patients with non-critical PAD who undergo minor limb amputations using CCTA and CACS and to examine their predictive value on 1-year all-cause mortality and MACEs, respectively compared to RCRI.

## Materials and methods

### Design and data sources

In this prospective observational cohort study, we present a unique cohort of 199 consecutive patients (mean age 62.3 ± 7.2 years; male 62.8%) with T2D and non-critical PAD undergoing PFA in the Republican Centre of Purulent Surgery and Complications of Diabetes, Tashkent Medical Academy, Tashkent, Uzbekistan and evaluate the predictive value of CACS and CCTA for MACE and 1-year all-cause mortality. Patients were without previous history of major or minor limb amputations, had no contraindications and were eligible to CCTA. Patients were followed every week after discharge during the first month, and every 1–2 months during 1-year follow-up. Of overall 230 patients underwent CCTA, 31 of these patients could not be contacted beyond one week after surgery, and 199 patients had 1-year follow-up data and included in the analysis.

### Data acquisition

Baseline characteristics were retrieved on the day of the admission to the hospital. Patient’s risk factors such as obesity, hypertension, history of cardiovascular disease (CVD), history of diabetes were assessed along with socio-economic factors, lifestyle risk factors such as self-reported smoking status, physical activity, and nutrition. Diabetes was defined by a hemoglobin HbA1C ≥ 6.5%, history of physician based diagnosis, or use of anti-diabetic medications according to 2019 ESC Guidelines on diabetes, pre-diabetes, and cardiovascular diseases.^2^ Smoking was defined as current (tobacco products used within the last month), occasional or never.^15^ Coronary artery disease was defined according to the 2019 ESC Guidelines for the diagnosis and management of chronic coronary syndromes.^15^ In adults (age over 18 years) obesity was defined by a BMI ≥ 30 kg/m.^16^ Blood pressure (BP) was measured after some rest, in sitting position, at the beginning and at the end of the healthcare providers exam in both arms, the mean of two measurements was used.^17,18^ Hypertension was defined according to guidelines of the Eighth Joint National Committee (JNC8).^17^

All patients had diagnosed T2D and also documented non-critical PAD with an ankle-brachial index (ABI) of < 0.9 of the affected extremity which required forefoot PFA.^19^ Foot ulcer and/or gangrene were classified as stage C (ischemia) or stage D (ischemia and infection), and grade 2–3 (deep and very deep) according to the Texas Wound Classification or grade 4 and 5 according to Wagner – Meggitt’s classification.^20^ There were no previous minor or major amputations of any extremity prior current hospitalization. All patients included in the analysis had creatinine levels within normal limits to be eligible for CCTA.

Fuster BEWAT score was calculated based on patients’ BP, weight and BMI, and history of physical activity per week, alimentation, and tobacco smoking (current smoker with counting cigarettes per day, occasional smoker, ex-smoker, never smoker).^21^ Charlson comorbidity index was also assessed.^22^

### Coronary artery calcium score and CCTA

Coronary artery calcium score-scoring (CACS) was performed on the day of evaluation immediately before CCTA. The protocol has previously been published.^5,23^ Based on the CACS (Agatston), patients were divided into five categories: 0, 0–99, 100–399, 400–999, ≥ 1000.^24^

Coronary artery atherosclerosis was classified as no stenosis (no-CAD), non-obstructive stenosis with luminal diameter narrowing < 50% (non-obstructive CAD), or obstructive stenosis with ≥50% artery obstruction.^23^ Single-vessel obstructive CAD was defined if there was a stenosis > 50% in one vessel; 2-vessel disease if there was stenosis > 50% in two major vessels, and 3-vessel obstructive CAD if there was stenosis > 50% in three major coronary arteries or in the left main artery.^23^

The model CCTA and CACS was compared with the model based on the revised cardiac risk index alone. The revised cardiac risk index (RCRI) was calculated for each patient. The RCRI relies on the presence or absence of the following five identifiable predictive factors: ischemic heart disease, congestive heart failure, cerebrovascular disease, insulin therapy with diabetes mellitus, and renal dysfunction (serum creatinine level > 2.0 mg/dl). One point was assigned for each of these predictors.^25^ The pooled risk estimates of external validation studies of the RCRI during past 15 years, showed risk estimates for myocardial infarction (MI), cardiac arrest, or death of 3.9% (95% CI, 2.8%–5.4%) for an RCRI score of 0, 6.0% (95% CI, 4.9%–7.4%) for an RCRI score of 1, 10.1% (95% CI, 8.1%–12.6%) for an RCRI score of 2, and 15.0% (95% CI, 11.1%–20.0%) for an RCRI score > 3.^26^

### Cardiovascular outcomes and all-cause mortality

Clinical end-points in this study were all-cause mortality and the occurrence of MACE, which were defined as a composite endpoint of ischemic endpoints as cardiovascular death, nonfatal MI, stent thrombosis, acute stroke, or unstable arrhythmia.^27^ Acute stroke was diagnosed based on clinical presentation and non-contrast brain CT.^27,28^ Cardiovascular death was defined as death due to MI, congestive heart failure, stroke, or arrhythmias or any unknown causes of death not explained by non-cardiac etiologies. Diagnosis of MI was confirmed by two of three findings: chest pain or equivalent symptom complex; positive cardiac biomarkers; ECG changes typical of MI.^27,29^ In patients who had multiple cardiac events, only the first one was counted toward MACE to create the MACE-free Kaplan-Meyer curve. Follow-up cardiac events were collected by scheduled appointments, chart review, telephone interview, and confirmation by social death certificate.

### Statistical analysis

Statistical analyses were performed using SPSS software (v27, IBM, Chicago, IL, USA). Descriptive statistics for studied variables are presented as mean ± SD (standard deviation) for normally distributed continuous variables, median with interquartile range for non-normally distributed continuous variables and frequency with percentage for categorical variables. Variables were compared with independent Student t tests for normally distributed continuous data, and Chi-square test for categorical data. Differences between groups were determined by a one-way analysis of variance (ANOVA), with a subsequent Tukey’s/Dunnet C post hoc test.

Scatter and box plots were used to visualize values calculated from the established equation in the SPSS software, box plots over dot plots were created in the “R” software to represent the values of the individual results as dots with the boxplot displaying the distribution of data. Time to event was calculated as the period between the CCTA study and death or MACE. Unadjusted Kaplan–Meier curves for MACE-free and all-cause mortality free survival were created depending on CACS and CCTA groups using the log-rank test (Mantel-Cox) along with Breslow (Generalized Wilcoxon) and Tarone-Ware tests for the period from the PFA during 1-year follow-up.

The association of RCRI, CACS and CCTA parameters with the risk of all-cause mortality and incident MACE was assessed using Cox proportional hazard models. A *p*-value of < .05 was considered statistically significant. Variables used in the analysis did not have missing data. The receiver operating characteristic curve (ROC) and area under curve (AUC) were used to evaluate the predictive value of the CCTA and CACS models compared to RCRI on the risk of all-cause mortality and incidence of MACE. DeLong’s test was used to observe AUC differences in the models. The Delong test was used to compare the performance among models using MedCalc software Version 20.113–64-bit. Reverse cardiovascular risk index have previously shown high predictive value for 1-year all-cause mortality for PAD patients undergoing limb vascular surgeries.^13^

## Results

Table 1 summarizes baseline patient characteristics of 199 patients who underwent CCTA and CACS. Overall cohort of patients undergoing minor limb amputation had uncontrolled diabetes (HbAC1, 11.4 ± 2.6%). Peripheral artery disease was non-critical. Systolic ankle pressure on the extremity required PFA was greater 110 mmHg, and diastolic over 60 mmHg, ankle-brachial pressure was ranged between the lowest 0.64, and highest 0.88. Great toe amputation was performed in 54 patients (27.1%), one toe or a combination of 2–5 toes in 82 (41.2%), and trans metatarsal amputation in 49 patients (24.6%). There was no association observed regarding the level of foot amputation and mortality.

**Table 1.** Baseline characteristics of patients with type 2 diabetes and peripheral artery disease undergoing partial foot amputation.

Baseline characteristic	CCTA and CACS *n* = 199
Age (years), mean ± SD	62.3 ± 7.2
Gender, *n* (%)	
Male	125 (62.8)
Female	74 (37.2)
Smoker status, *n* (%)	
Smoker > 1pack/day	17 (8.5)
Smoker < 1pack/day	62 (31.2)
Occasional smoker	34 (17.1)
Non-smoker	86 (43.2)
BMI (kg/m^2^), mean ± SD	28.7 ± 3.9
Normal weight, *n* (%)	53 (13.2)
Overweight, *n* (%)	214 (53.4)
Obesity, *n* (%)	134 (33.4)
Aspirin before hospitalization, *n* (%)	135 (67.8)
Lipid-lowering drugs before hospitalization, *n* (%)	42 (21.1)
New diagnosed diabetes, *n* (%)	11 (5.5)
Blood glucose, mean ± SD	12.2 ± 4.3
HbAC1, %, mean ± SD	11.4 ± 2.6
Arterial hypertension, *n* (%)	115 (57.8)
Clinical coronary artery disease, *n* (%)	153 (76.9)
Symptomatic typical	51 (25.6)
Symptomatic atypical	78 (39.2)
Asymptomatic	24 (12.1)
Previous myocardial infarction, *n* (%)	55 (27.6)
Reverse cardiac risk index, *n* (%)	
II	45 (22.6)
III	58 (29.1)
IV	96 (48.2)
Fuster-BEWAT score, mean ± SD	6.3 ± 2.1
Charlson comorbidity score, mean ± SD	6.4 ± 1.8

BMI = body mass index, SD = standard deviation.

Of 199 patients who underwent CCTA, 27 (13.6%) did not have coronary stenosis, whereas 172 patients (86.4%) had CAD, of them 77 patients (38.7%) with non-obstructive stenosis<50%, and 95 patients (47.7%) had obstructive CAD. Patients without coronary stenosis on CCTA (*n* = 27) were younger with a mean age 46.4 ± 6.5 years, 11/27 patients had newly diagnosed diabetes, and 16/27 patients had a history of diabetes of less than 2 years.

### The risk of all-cause mortality

**Figure 1.**
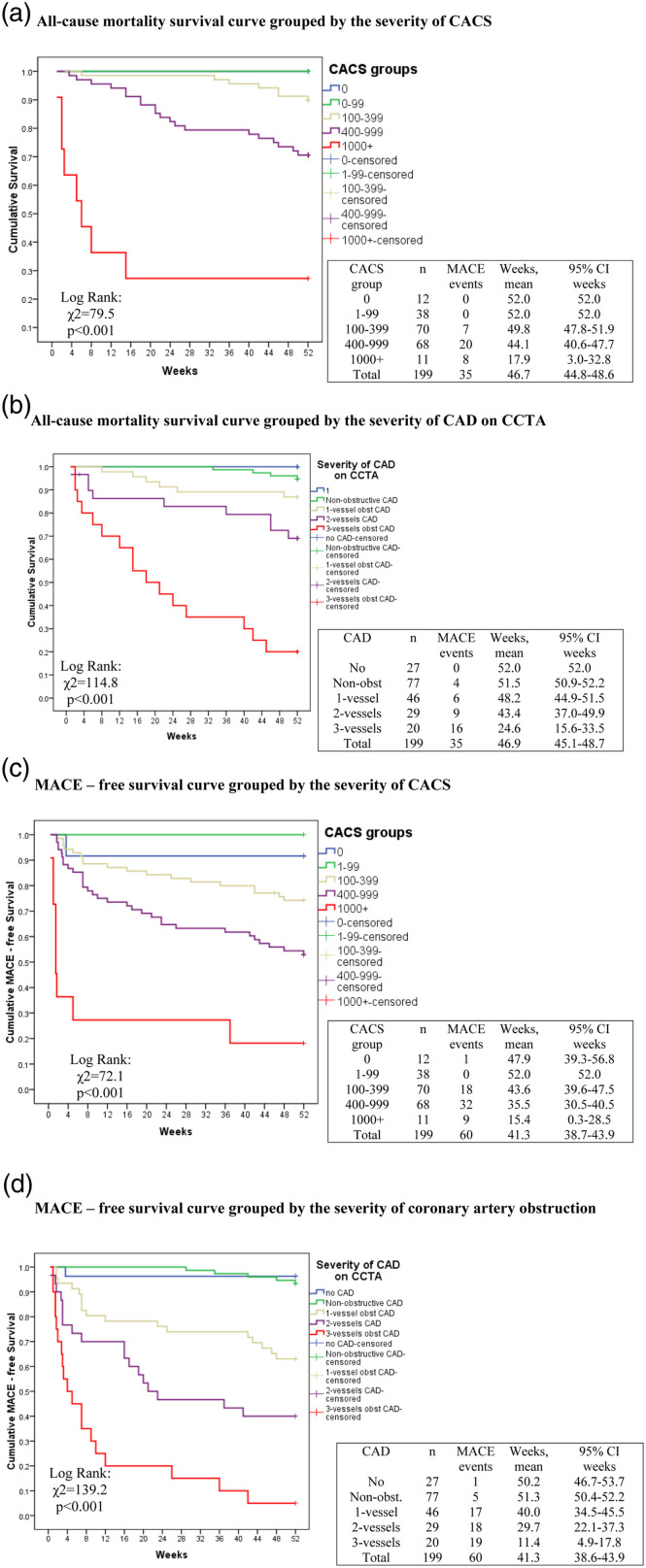
Kaplan-Meier survival curves for all-cause mortality and incidence of major adverse cardiovascular events (MACE) in patients with type 2 diabetes undergoing partial foot amputation stratified by coronary artery calcium score (CACS) and results of coronary computed tomographic angiography (CCTA). Coronary artery disease (CAD) on CCTA was classified as no stenosis (no-CAD), non-obstructive CAD (luminal diameter narrowing <50%), or 1, 2, 3-vessels obstructive CAD with ≥50% artery obstruction. CAD – coronary artery disease, CACS – coronary artery calcium score, CCTA – coronary computed tomographic angiography, MACE – major adverse cardiovascular events, CI – confidence interval.

There was no in-hospital mortality and 7 days after hospital discharge. 30-days all-cause mortality was 6/199 (3.0%), 1-year mortality was 35/199 (17.6%). One-year all-cause mortality-free Kaplan-Meier survival curves grouped by the severity of CACS are shown in Figure 1(a). With increased CACS, 1-year all-cause mortality increased from zero in CACS = 0–99, 10% (CACS = 100–399), 29.5% (CACS = 400–999), to 72.8% (CACS ≥ 1000) (Figure 1(a)).

One-year all-cause mortality-free Kaplan-Meier survival curve grouped by the severity of coronary arteries obstruction on CCTA is shown in Figure 1(b). Patients with two- and three- vessels obstructive CAD had higher all-cause mortality compared to no-CAD, non-obstructive CAD, 1-vessel obstructive CAD (*p* < .05). There were no statistically significant differences between no-CAD, non-obstructive CAD, and 1-vessel obstructive CAD groups (Figure 1(b)).

Univariable and multivariable Cox regression to examine the association between RCRI, CACS and coronary arteries obstruction with the risk of 1-year all-cause mortality in T2D patients undergoing PFA is shown in Table 2. After adjusting for confounders, both CACS and severity of coronary arteries obstruction (measured by CCTA) was associated with increased risk of all-cause mortality**.** Compared to no-stenosis on CCTA (reference), the 1-year all-cause mortality in non-obstructive CAD increased (HR = 1.38, 95% CI [0.75–12.86], *p* = .284), 1-vessel obstructive CAD (HR = 8.13, 95% CI [0.87–75.88], *p* = .066), 2-vessels obstructive CAD (HR = 10.94, 95% CI [1.03–115.8], *p* = .047), and 3-vessels obstructive CAD (HR = 45.73, 95% CI [4.6–454.7], *p* = .001) **(**Table 2**).**

**Table 2.** Cox regression analysis to determine the association of RCRI, CACS, and CCTA with the risk of 1-year all-cause mortality and major adverse cardiovascular events, respectively, in type 2 diabetes patients undergoing partial foot amputation.

	Unadjusted		Adjusted	
All-cause mortality	HR (95% CI)	*p* Value	HR (95% CI)	*p* Value
RCRI				
II	1		1	
III	1.75 (1.05, 2.94)	.032	2.26 (0.9, 5.69)	.084
IV	3.09 (1.95, 4.91)	<.001	4.17 (0.88, 19.67)	.071
CACS	1.003 (1.002, 1.004)	<.001	1.002 (1.000, 1.003)	.061
CCTA				.001
No CAD	1		1	
Non-obstructive	1.75 (0.2, 14.9)	.611	1.38 (0.75, 12.86)	.284
1-Vessel obstructive	11.9 (1.59, 89.7)	.016	8.13 (0.87, 75.88)	.066
2-Vessels obstructive	25.5 (3.39, 191.3)	.002	10.94 (1.03, 115.8)	.047
3-Vessels obstructive	77.5 (10.3, 585.5)	<.001	45.73 (4.60, 454.7)	.001
Major adverse cardiovascular events	HR (95% CI)	*p* Value	HR (95% CI)	*p* Value
RCRI				
II	1		1	
III	1.82 (0.92, 3.57)	.084	1.12 (0.27, 4.59)	.874
IV	3.66 (2.0, 6.68)	<.001	0.59 (0.11, 3.08)	.531
CACS	1.004 (1.003, 1.005)	<.001	1.002 (1.001, 1.003)	.013
CCTA				<.001
No CAD	1		1	
Non-obstructive	1.74 (0.2, 14.9)	.611	1.59 (0.75, 12.77)	.124
1-Vessel obstructive	11.9 (1.6, 89.9)	.016	5.74 (1.89, 17.38)	.002
2-Vessels obstructive	24.2 (3.2, 182.3)	.002	7.92 (2.29, 27.26)	.001
3-Vessels obstructive	79.2 (10.5, 598.2)	<.001	32.85 (9.77, 110.4)	<.001

Adjustment to age, sex, HbAC1, Fuster-BEWAT score, Charlson comorbidity, RCRI, symptomatic CAD, prior MI, prior coronary revascularization.

CAD = coronary artery disease, CACS = coronary artery calcium score, CCTA = coronary computed tomographic angiography, HR = hazard ratio, RCRI = revised cardiac risk index.

**Figure 2.**
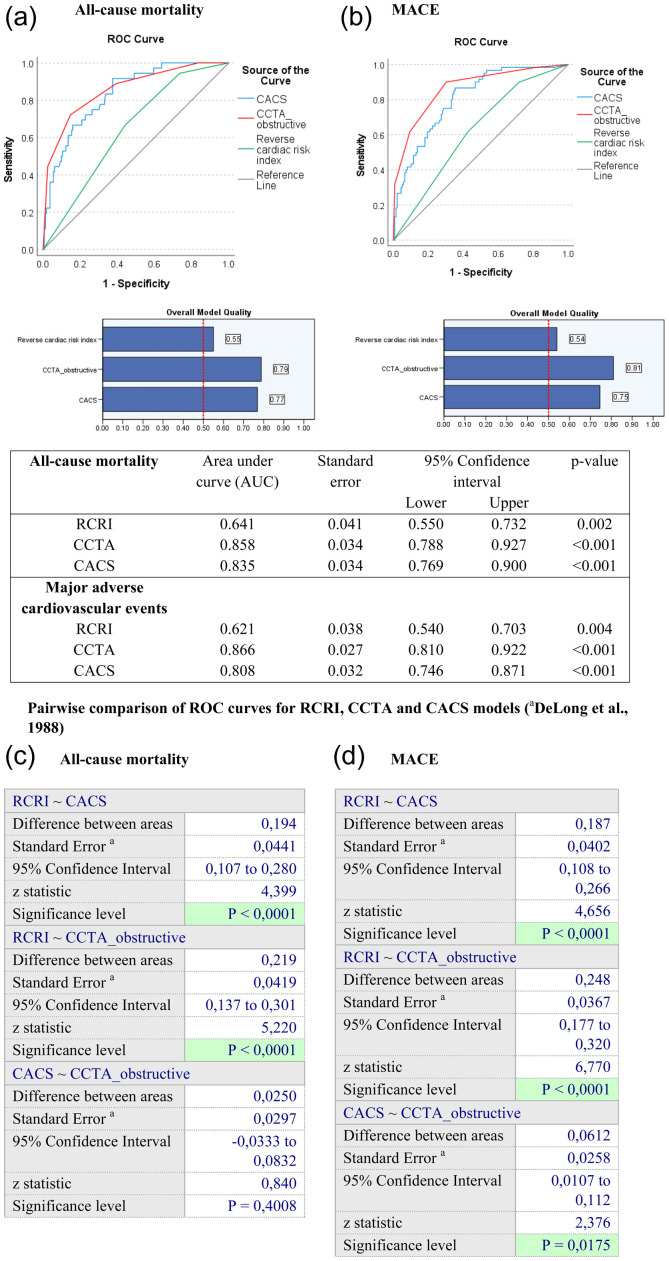
The receiver operating characteristic curve (ROC) and area under curve (AUC) to evaluate the predictive value of the coronary computed tomographic angiography (CCTA) and coronary artery calcium score (CACS) models compare to revised cardiac risk index (RCRI) on all-cause mortality (A) and incidence of major adverse cardiovascular events (B) in type 2 diabetes patients and non-critical peripheral artery disease undergoing partial foot amputation. Pairwise comparison of ROC curves for RCRI, CCTA and CACS models for all-cause mortality (C) and incidence of major adverse cardiovascular events (MACE) (D) (^a^DeLong et al., 1988).

The AUC of ROC curve analysis for CACS and mortality was 0.835 (95% CI: 0.769–0.900, *p* ≤ .001), for CCTA and mortality 0.858 (95% CI: 0.788–0.927, *p*≤ .001). The control RCRI model (score 4 and above) showed AUC 0.641 (95% CI: 0.550–0.7323, *p* = .002). Delong test showed that the RCRI model, CACS and CCTA COX models were statistically significant. The CACS and CCTA models markedly improved prediction performance for all-cause mortality (*p* < 0.0001), adding difference of 0.2 between areas to RCRI model (Figure 2(a) and (c)).

**Figure 3.**
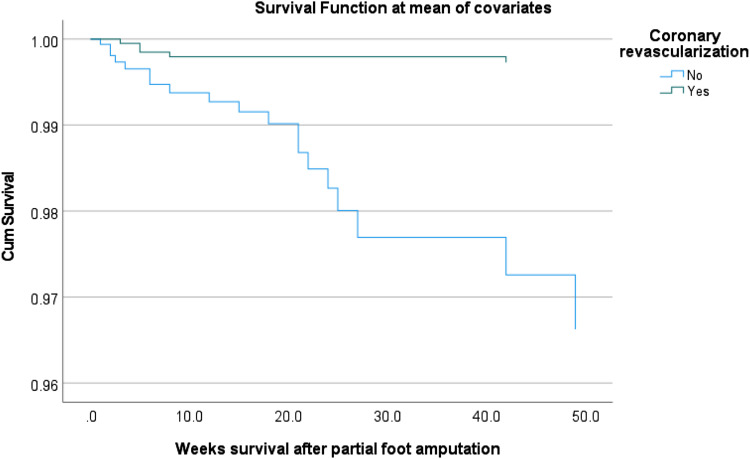
Cox regression survival curve in patients with type 2 diabetes and peripheral artery disease undergoing partial foot amputation stratifying by coronary revascularization during 1-year follow up. Log rank test (*p* = .001) showed significantly improved survival in patients who underwent coronary revascularization compared with patients without revascularization.

61/95 patients with obstructive CAD underwent coronary revascularization, the decrease in cardiovascular mortality was OR = 0.03 (CI95% 0.005; 0.17), *p* < 0.001 compared to patients without heart revascularization, after adjustment for CCTA, CACS, baseline characteristics. Of 30 patients who had obstructive CAD and died, eight patients underwent coronary revascularization (1/6 1-vessel, 2/9 2-vessels, and 5/15 3-vessels disease). However, three out of 8 deaths were not cardiovascular related (1 due to gastro-intestinal bleeding, one due to massive pulmonary embolism, one due to sepsis). Cox regression survival curve log rank test (*p* = .001) showed significantly improved survival in patients who underwent coronary revascularization during 1-year follow-up when the obstructive coronary artery disease was detected on CCTA compared with patients without revascularization (Figure 3).

**Figure 4.**
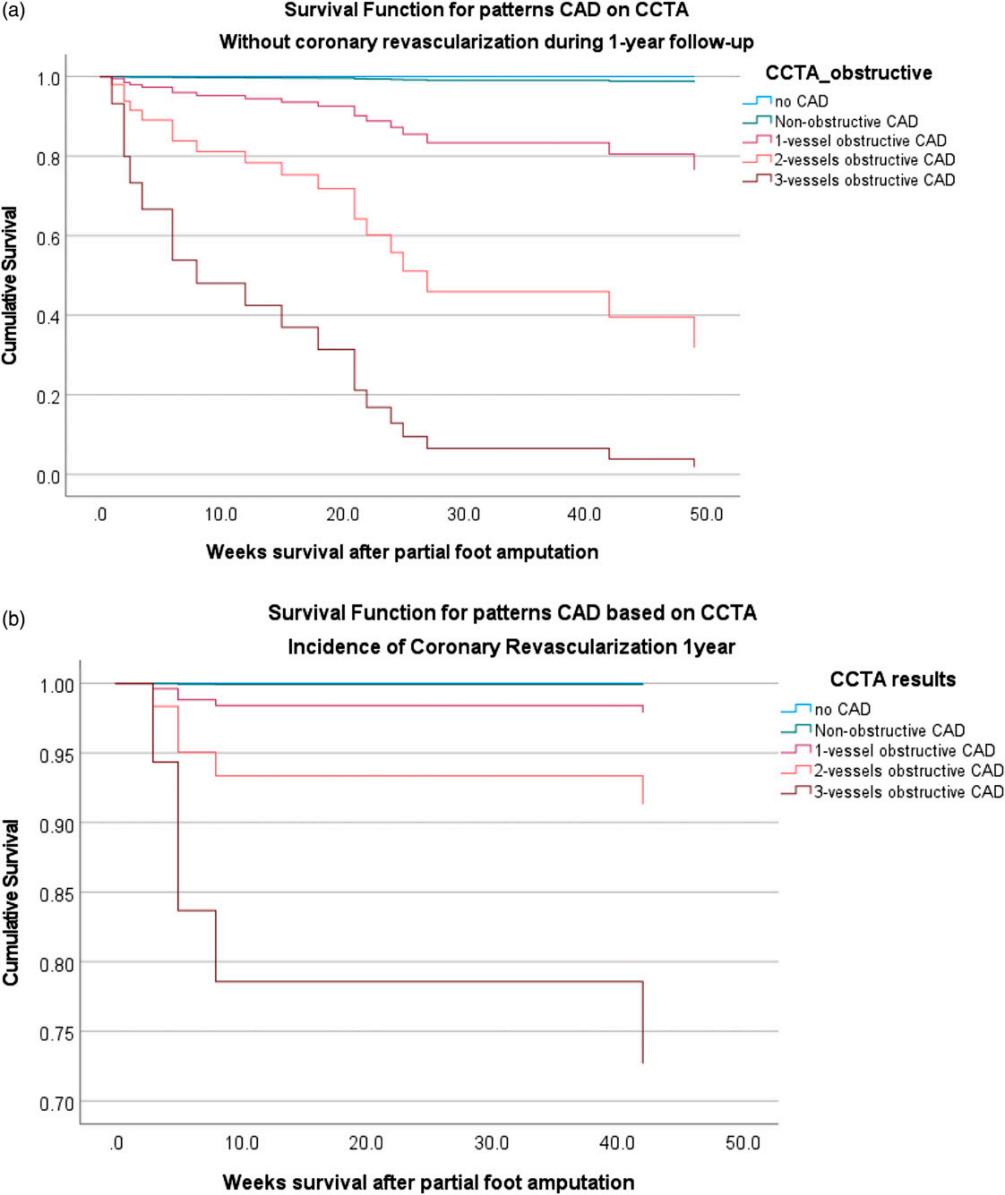
A Cox proportional-hazard regression analysis to assess the association between the severity of coronary artery obstruction and 1-year mortality stratified by history of coronary revascularization performed during 1-year follow-up, after adjustment for baseline variables (A) no coronary revascularization performed, (B) with heart revascularization.

A Cox proportional-hazard regression analysis was performed to assess association between severity of coronary obstruction detected on CCTA and 1-year mortality, stratified by weather or not coronary revascularization was performed during 1-year follow-up. In overall cohort (*n* = 199), Cox regression analysis showed that coronary revascularization was associated with decreased 1-year all-cause mortality (HR 0.4 with 95% CI 0.3–0.6, *p* .001) after adjustment for baseline variables (Figure 4).

### The incidence of MACE

One-year incidence of MACE was 30.2% (60/199 patients). Unadjusted 1-year MACE-free Kaplan-Meier survival curve grouped by the severity of CACS is shown in Figure 1(c). Within 1-year follow-up, MACE was observed in 2% of patients (CACS = 0–99), 25.8% (CACS = 100–399), 47.1% (CACS = 400–999), to 81.9% (CACS ≥ 1000). Unadjusted MACE–free Kaplan-Meier curve grouped by severity of coronary artery obstruction on CCTA is shown in Figure 1(d). There was a significant difference between patients with no-CAD, non-obstructive CAD, 1-vessel obstructive CAD compared to the two- and three- vessels obstructive CAD (*p* < .05).

Results from univariable and multivariable Cox regression analysis to examine the association between CACS and severity of coronary artery obstruction with 1-year incidence of MACE in T2D patients undergoing PFA is shown in Table 2. After adjusting for age, sex, HbAC1, Fuster-BEWAT, Charlson comorbidity, RCRI, symptomatic CAD, prior MI, prior coronary revascularization, both CACS and CCTA significantly increased HR of 1-year incidence of MACE (Table 2). There was no statistical difference in the incidence of MACE between patients with no coronary stenosis and patients with mild non-obstructive CAD (HR = 1.59, 95% CI [0.75, 12.77], *p* = .124). Compared to no stenosis (reference), having 1-vessel obstructive CAD increased incidence of MACE 5.74-fold (HR = 5.74, 95% CI [1.89, 17.38], *p* = .002), having 2-vessels obstructive CAD approximately 8-fold (HR = 7.92, 95% CI [2.29, 27.26] *p* = .001), and having 3-vessels obstructive CAD 32-fold (HR = 32.85, 95% CI [9.77, 110.4], *p* < .001) (Table 2). The AUC of ROC curve analysis for the CACS and MACE was 0.808 (95% CI: 0.746–0.871, *p* ≤ .001), for CCTA and MACE 0.866 (95% CI: 0.810–0.922, *p* ≤ .001). The control RCRI model showed AUC 0.621 (95% CI: 0.540–0.703, *p* = .004). Delong test showed that the RCRI model, CACS and CCTA COX models were statistically significant, and the CACS and CCTA models significantly improved prediction performance for the incidence of MACE compared with RCRI model (*p*  < .0001). Coronary computed tomographic angiography model showed statistically significant superiority over CACS model (difference between areas 0.0612, 95% CI 0107 to 0.112; *p* = .0175) (Figure 2(b) and (d)).

#### Additional tests

The distribution of the CACS among patients depending on the degree of coronary artery obstruction on CCTA is shown in the Supplement 1. The ANOVA between groups was significant, F (4, 194) = 68.8, *p* ≤ .001. There was a significant pairwise difference between the group with no CAD as well as with non-obstructive CAD in between all obstructive CAD groups (*p* ≤ .001 for all pairwise combinations). There was also a significant difference between 1-vessel obstructive CAD and two- and three-vessels obstructive CAD. The mean differences were 156.5 ± 45.2 (*p* = .006), and 187.5 ± 51.5 (*p* = .003), respectively. However, there was no significant difference of CACS values between 2-vessels and 3-vessels obstructive CAD. CACS was positively related to the increase of obstruction of coronary arteries, r = 0.74, *p* < .001. The variance of the CAD obstruction which can be explained by CACS is 55.2%.

One-year mortality in patients depending on the CACS and the severity of the coronary obstruction on CCTA and are shown in the Supplement 2. Approximately 70% deaths in patients with obstructive CAD were associated with MACE.

The severity of coronary stenosis using an 18-segmental model on CCTA is shown in the Supplement 3. Patients undergoing PFA had more pronounced distant coronary arteries obstruction versus proximal parts of vessels.

## Discussion

The novelty of the study consists in the utilization of a prediction model using a RCRI as a reference to show the added value for CCTA and CACS to predict 1-year all-cause mortality. Our results indicated that severity of CAD shown in preoperative CACS and CCTA in patients with T2D and non-critical PAD who undergo minor foot surgery such as amputation of toes or trans-metatarsal, have a high predictive value for short-term postoperative cardiovascular events and all-cause mortality. The AUC of ROC curve analysis to predict 1-year all-cause mortality for CACS and CCTA had added value and significantly improved the control model RCRI. Similar findings showed prediction of MACE for the CACS and CCTA compared with the control RCRI model.

Our findings underline the importance to assess CAD preoperatively in these patients in particular in view of the fact that the short-term risk for serious cardiovascular complications and mortality risk may often be underestimated in these patients. In addition to local foot care and eventually also to limb revascularization, coronary revascularization might be indicated if extensive obstructive CAD is present. Our findings are of particular importance for countries with economy in transition where there is limited access to catheterization labs and appropriate patient selection for coronary interventions is mandatory.

Preoperative evaluation of the presence and extension of CAD and timely coronary revascularization is important to decrease the perioperative risk and improve the postoperative long-term prognosis by choosing optimal treatment strategies. Exercise ECG testing, stress myocardial perfusion scintigraphy, or stress echocardiography for the detection of obstructive CAD are often contraindicated in this type of patients because of lower limb disabilities and limb infection requiring emergency surgery.^5,6,7^ Over one-third of perioperative MACE occurred in patients with negative results of such testing.^30^

In real world clinical practice, patients with non-critical PAD and unhealed deep foot ulcer or toes gangrene which require minor foot amputation, are under care of vascular/endovascular surgeons who are performing endovascular intervention, and commonly only focusing on the extremity target lesions without concerning of CAD evaluation and management.^11^ In general, minor amputations like PFA are not considered as high-risk surgery.^3,4^ However, 3-year mortality after below-knee amputations in T2D patients is estimated to be close to 40–60%, with most deaths attributed to CAD.^14^ Five-year mortality with any amputation (major and minor combined) is ranging from 53% to 100%.^31^ This rate did not change over decades despite of advanced limb revascularization techniques, and decreasing proportion of major amputations.^10^

It is well known that obstructive CAD has been observed in 54%–69% of patients with critical PAD.^11,12^ Up to 20% mortality rates within 6 months have been reported for critical PAD from diagnosis with the excess death of 50% at 5 years.^9^ In a recently published paper, Choi and co-authors showed, that of 674 patients with critical PAD and a history of limb percutaneous transluminal angioplasty underwent routine coronary angiography, 61% had obstructive CAD and subsequently performed coronary revascularization.^11^ Routine coronary angiography and subsequent percutaneous interventions resulted in similar long-term survival compared to those who did not have coronary artery disease.^11^

In our previous study in patients with T2D undergoing transfemoral amputations, the major cause of amputation was critical limb ischemia due to severe PAD. Coronary computed tomographic angiography showed a high prevalence of obstructive CAD and a very high incidence of MACE and mortality.^5,32^ There were more post-operative events in patients with three- and two-vessels compared to 1-vessel obstructive and non-obstructive CAD (74.1% and 34.1% vs. 10.5% and 6.5%, *p* < .001) (*p* < .001).^5,32^ In contrast, patients with non-critical limb ischemia undergoing minor foot surgery are usually not considered to be patients with a high perioperative cardiovascular risk and therefore interventions to prevent cardiovascular complications are usually not considered to be of high priority. Our new finding is that patients undergoing minor foot amputation are also at high risk for MACE and all-cause mortality which is not inferior compared to major limb amputations. This indicates a strong need for cardiovascular interventions to improve short-term prognosis and survival in most of these patients.

Optimal patient treatment relies on both the diagnostic and the prognostic information provided by noninvasive testing. Due to high costs which are associated with the use of advanced imaging methods and limited availability of highly sophisticated technical equipment in many countries, there has been a significant shift from focusing on test accuracy to a broader emphasis on patient outcome.^33^ Diabetic patients with complications requiring PFA are usually under surgeon’s care with a main focus on the wound healing process and lower extremity revascularization procedures. In this context, the use of CCTA and CACS for perioperative risk assessment may open new windows for effective strategies to improve long-term outcome and secondary/tertiary prevention.

Our results are in line with results of a recent randomized controlled trial, where Sharma and co-authors (2019) showed that in patients with diabetes and suspected CAD, CCTA may be considered as the initial diagnostic test versus functional stress testing.^34^ Among 1908 diabetic patientеs with randomly assigned tests, patients who underwent CCTA had a lower incidence of cardiovascular death/MI compared with functional stress testing (CCTA: 1.1% [10 of 936] vs. stress testing: 2.6% [25 of 972]; adjusted hazard ratio: 0.38).^34^

The relative risk of 1-year all-cause mortality among those who did not have coronary revascularization was 2.85 times greater than those who underwent revascularization (*p* = .006), and 4.33 times greater to die from cardiovascular disease (*p* < .001). These findings underline again the importance of preoperative assessment for coronary artery atherosclerotic lesions to allow timely coronary revascularization in these patients if indicated.

In Uzbekistan, there is no mandatory insurance coverage and patients have to pay out-of-pocket for the majority of medications, hospital treatment, and interventions. The costs for diabetes treatment including in-hospital surgery for amputations and all emergency services are included in the Guaranteed Benefits Package and covered by the government. However, patients had to cover costs for cardiovascular management by themselves which means a trade-off between high out-of-pocket costs for a priori heart revascularization versus receiving interventional treatment for free when MI already happened. Furthermore, diabetic polyneuropathy is a cause of asymptomatic CAD which made it hard for patients to believe that their heart had as severe problems as the presence of foot gangrene/infection, which is visible. Social protection and sick leave for patients with disabilities are poorly developed in Uzbekistan, meaning that limb loss will be more likely associated with job loss and social isolation and patients tried to focus on wound healing and restoring walking function after minor amputation. 18 of 30 patients with obstructive CAD could either not afford preventive coronary revascularization, decided to postpone heart revascularization prioritizing limb wound healing, or refused further cardiovascular interventions.

A strength of this study is that data comes from a unique relatively large cohort of consecutive patients who suffered from T2D, non-critical PAD and had to undergo PFA with no loss of 1-year follow-up. Our findings can help to improve in-hospital and early post-operative patients’ care, which is usually conducted by surgeons to control wound-healing process but should also include coronary imaging and assessment by a cardiologist. Such an approach has a great potential to reduce cardiovascular complications and to increase overall long-term survival after surgery in these high-risk patients.

### Study limitations

In this study, CCTA and CACS were performed in only 59% of patients who were eligible for CCTA. The major reason for not undergoing CCTA was that it was not possible for many patients to pay out of pocket for an additional expensive test before PFA surgery, sometimes combined with a personal negative attitude towards the prognostic testing. However, there was no significant difference in the baseline characteristics between the study patients and the entire patient cohort. Patients at the baseline had uncontrolled diabetes with a mean HbA1c of 11.4%, which indicates that the study results cannot be generalized and transferred to patients with well-controlled diabetes. SGLT-2i and GLP-1-RA are new to Uzbekistan and non-affordable for the majority of patients to purchase out-of-pocket. In the current study, none of patients used these types of medications to control blood glucose.

## Conclusion

Severity of CAD detected by CACS and CCTA have a high predictive value for 1-year MACE and all-cause mortality in T2D patients undergoing PFA and may be considered for perioperative risk assessment allowing timely preventive interventions if stress tests are not feasible and more sophisticated technical equipment is not available.

## Supplemental Material

Supplemental Material - Coronary artery calcium score and coronary computed tomography angiography predict one-year mortality in patients with type 2 diabetes and peripheral artery disease undergoing partial foot amputation Click here for additional data file. Supplemental Material for Coronary artery calcium score and coronary computed tomography angiography predict one-year mortality in patients with type 2 diabetes and peripheral artery disease undergoing partial foot amputation by Evgeniya Shalaeva, Arjola Bano, Ulugbek Kasimov, Bakhtiyor Janabaev, Iris Baumgartner, Markus Laimer and Hugo Saner in Diabetes and Vascular Disease Research

## References

[1] Nakazato R Arsanjani R Achenbach S, et al. Age-related risk of major adverse cardiac event risk and coronary artery disease extent and severity by coronary CT angiography: results from 15 187 patients from the international multisite CONFIRM study. Eur Heart J Cardiovasc Imaging 2014; 15: 586–594. 24714312 10.1093/ehjci/jet132 PMC3979454

[2] Cosentino F Grant PJ Aboyans V, et al. 2019 ESC guidelines on diabetes, pre-diabetes, and cardiovascular diseases developed in collaboration with the EASD. Eur Heart J 2020; 41: 255–323. 31497854 10.1093/eurheartj/ehz486

[3] Schwarze ML Barnato AE Rathouz PJ, et al. Development of a list of high-risk operations for patients 65 years and older. JAMA Surg 2015; 150: 325–331. 25692282 10.1001/jamasurg.2014.1819 PMC4414395

[4] Olin JW Sealove BA. Peripheral artery disease: current insight into the disease and its diagnosis and management. In: Mayo Clinic Proceedings. Netherlands: Elsevier Ltd, pp. 678–692. 10.4065/mcp.2010.0133 PMC2894725 20592174

[5] Shalaeva E V Saner H Janabaev BB, et al. Coronary artery calcium score and coronary computed tomographic angiography for major perioperative cardiovascular complications in symptomatic diabetic patients undergoing trans-femoral amputation. Int J Cardiol 2016; 221: 806–811. 27428325 10.1016/j.ijcard.2016.06.165

[6] Kristensen SD Knuuti J Saraste A, et al. 2014 ESC/ESA guidelines on non-cardiac surgery: cardiovascular assessment and management: the joint task force on non-cardiac surgery: cardiovascular assessment and management of the European society of cardiology (ESC) and the European society of anaesthesiology (ESA). Eur Heart J 2014; 35: 2383–2431. 25086026 10.1093/eurheartj/ehu282

[7] Aboyans V Ricco JB Bartelink MLEL, et al. 2017 ESC guidelines on the diagnosis and treatment of peripheral arterial diseases, in collaboration with the European society for vascular surgery (ESVS). Eur Heart J 2018; 39: 763–816.28886620

[8] Knight SR Ho A Pius R, et al. Risk stratification of patients admitted to hospital with covid-19 using the ISARIC WHO clinical characterisation protocol: development and validation of the 4C Mortality Score. BMJ 2020; 370: m3339. DOI: 10.1136/bmj.m3339 32907855 PMC7116472

[9] Teraa M Conte MS Moll FL, et al. Critical limb ischemia: current trends and future directions. *J Am Heart Assoc* 2016; 5: e002938. DOI: 10.1161/JAHA.115.002938 26908409 PMC4802465

[10] Jupiter DC Thorud JC Buckley CJ, et al. The impact of foot ulceration and amputation on mortality in diabetic patients. I: From ulceration to death, a systematic review. Int Wound J 2015; 13: 892–903. 25601358 10.1111/iwj.12404 PMC7950078

[11] Choi BG Hong JY Rha SW, et al. Long-term outcomes of peripheral arterial disease patients with significant coronary artery disease undergoing percutaneous coronary intervention. PLoS One 2021; 16: e0251542. DOI: 10.1371/JOURNAL.PONE.0251542 34010351 PMC8133421

[12] Lee MS Rha SW Han SK, et al. Clinical outcomes of patients with critical limb ischemia who undergo routine coronary angiography and subsequent percutaneous coronary intervention. J Invasive Cardiol 2015; 27: 213–217. 25840405

[13] Moitra VK Flynn BC Mazzeffi M, et al. Indication for surgery, the revised cardiac risk index, and 1-year mortality. Ann Vasc Surg 2011; 25: 902–908. 21820856 10.1016/j.avsg.2011.05.010

[14] Brownrigg JRW Apelqvist J Bakker K, et al. Evidence-based management of PAD & the diabetic foot. Eur J Vasc Endovascular Surg 2013; 45: 673–681. 10.1016/j.ejvs.2013.02.014 23540807

[15] Neumann FJ Sechtem U Banning AP, et al. 2019 ESC guidelines for the diagnosis and management of chronic coronary syndromes. Eur Heart J 2020; 41: 407–477. 31504439 10.1093/eurheartj/ehz425

[16] Yumuk V Tsigos C Fried M, et al. European guidelines for obesity management in adults. Obes Facts 2015; 8: 402–424. 26641646 10.1159/000442721 PMC5644856

[17] Bakris G Ali W Parati G. ACC/AHA Versus ESC/ESH on hypertension guidelines: JACC guideline comparison. J Am Coll Cardiol 2019; 73: 3018–3026. 31196460 10.1016/j.jacc.2019.03.507

[18] Williams B Mancia G Spiering W, et al. 2018 ESC/ESH guidelines for the management of arterial hypertension. Eur Heart J 2018; 39: 3021–3104. 30165516 10.1093/eurheartj/ehy339

[19] Rutherford RB Baker JD Ernst C, et al. Recommended standards for reports dealing with lower extremity ischemia: Revised version. J Vasc Surg 1997; 26: 517–538. 9308598 10.1016/s0741-5214(97)70045-4

[20] Frykberg RG. Diabetic foot ulcers: pathogenesis and management, www.aafp.org/afpAMERICANFAMILYPHYSICIAN1655 (1 November 2002, accessed 13 May 2021). 12449264

[21] Gómez-Pardo E Fernández-Alvira JM Vilanova M, et al. A comprehensive lifestyle peer group–based intervention on cardiovascular risk factors: the randomized controlled fifty-fifty program. J Am Coll Cardiol 2016; 67: 476–485. 26562047 10.1016/j.jacc.2015.10.033

[22] Charlson ME Pompei P Ales KL, et al. A new method of classifying prognostic comorbidity in longitudinal studies: Development and validation. J Chronic Dis 1987; 40: 373–383. 3558716 10.1016/0021-9681(87)90171-8

[23] Leipsic J Co-Chair F Abbara S, et al. SCCT guidelines SCCT guidelines for the interpretation and reporting of coronary CT angiography: a report of the society of cardiovascular computed tomography guidelines committee. J Cardiovasc Comput Tomogr 2014; 8: 342–358. 25301040 10.1016/j.jcct.2014.07.003

[24] Hecht HS Cronin P Blaha MJ, et al. 2016 SCCT/STR guidelines for coronary artery calcium scoring of noncontrast noncardiac chest CT scans: a report of the society of cardiovascular computed tomography and society of thoracic radiology. J Cardiovasc Comput Tomogr 2017; 11: 74–84. 27916431 10.1016/j.jcct.2016.11.003

[25] Piepoli MF Hoes AW Agewall S, et al. 2016 European guidelines on cardiovascular disease prevention in clinical practice. Eur Heart J 2016; 37: 2315–2381. 27222591 10.1093/eurheartj/ehw106 PMC4986030

[26] Duceppe E Parlow J Macdonald P, et al. Society guidelines Canadian cardiovascular society guidelines on perioperative cardiac risk assessment and management for patients who undergo noncardiac surgery. Can J Cardiol 2017; 33: 17–32. 27865641 10.1016/j.cjca.2016.09.008

[27] Hupfeld C Mudaliar S. Navigating the “MACE” in cardiovascular outcomes trials and decoding the relevance of atherosclerotic cardiovascular disease benefits versus heart failure benefits. Diabetes Obes Metab 2019; 21: 1780–1789. 30957945 10.1111/dom.13740

[28] Messerli FH Shalaeva EV Rexhaj E. Optimal BP targets to prevent stroke and MI: is there a lesser of 2 evils? J Am Coll Cardiol 2021; 78: 1679–1681. 34674812 10.1016/j.jacc.2021.09.013

[29] Merchant RM Topjian AA Panchal AR, et al. Part 1: executive summary: 2020 American heart association guidelines for cardiopulmonary resuscitation and emergency cardiovascular care. Circulation 2020; 142: S337–S357. 33081530 10.1161/CIR.0000000000000918

[30] Koshy AN Ha FJ Gow PJ, et al. Computed tomographic coronary angiography in risk stratification prior to non-cardiac surgery: a systematic review and meta-analysis. Heart 2019; 105: 1335–1342. 31018953 10.1136/heartjnl-2018-314649

[31] Thorud JC Plemmons B Buckley CJ, et al. Mortality after nontraumatic major amputation among patients with diabetes and peripheral vascular disease: a systematic review. J Foot Ankle Surg 2016; 55: 591–599. 26898398 10.1053/j.jfas.2016.01.012

[32] Shalaeva E V Saner H Janabaev BB, et al. Tenfold risk increase of major cardiovascular events after high limb amputation with non-compliance for secondary prevention measures. Eur J Prev Cardiol 2017; 24: 708–716. 28071959 10.1177/2047487316687103

[33] Fordyce CB Newby DE Douglas PS. Diagnostic strategies for the evaluation of chest pain clinical implications from SCOT-HEART and PROMISE. J Am Coll Cardiol 2016; 67: 843–852. 26892420 10.1016/j.jacc.2015.11.055 PMC4771619

[34] Sharma A Coles A Sekaran NK, et al. Stress testing versus CT angiography in patients with diabetes and suspected coronary artery disease. J Am Coll Cardiol 2019; 73: 893–902. 30819356 10.1016/j.jacc.2018.11.056 PMC7101508

