# Supplementary material for: Coronary artery calcium score and coronary computed tomography angiography predict one-year mortality in patients with type 2 diabetes and peripheral artery disease undergoing partial foot amputation
Source: Diab Vasc Dis Res. 2022 Oct 12;19(5):14791641221125190. doi: 10.1177/14791641221125190 (PMC9558880; doi:10.1177/14791641221125190)
Supplement: Supplemental Material - Coronary artery calcium score and coronary computed tomography angiography predict one-year mortality in patients with type 2 diabetes and peripheral artery disease undergoing partial foot amputation [file sj-pdf-1-dvr-10.1177_14791641221125190.pdf]

Supplement 1.

Distribution of the coronary artery calcium score (CACS) among patients depending on the severity of coronary artery disease based on coronary computed tomographic angiography. The number of independent patients is indicated by the individual circle data points and statistical analyses indicated by the box and wisker plot was determined using ANOVA with a subsequent LSD/Tukey's/Dunnet C post hoc test.

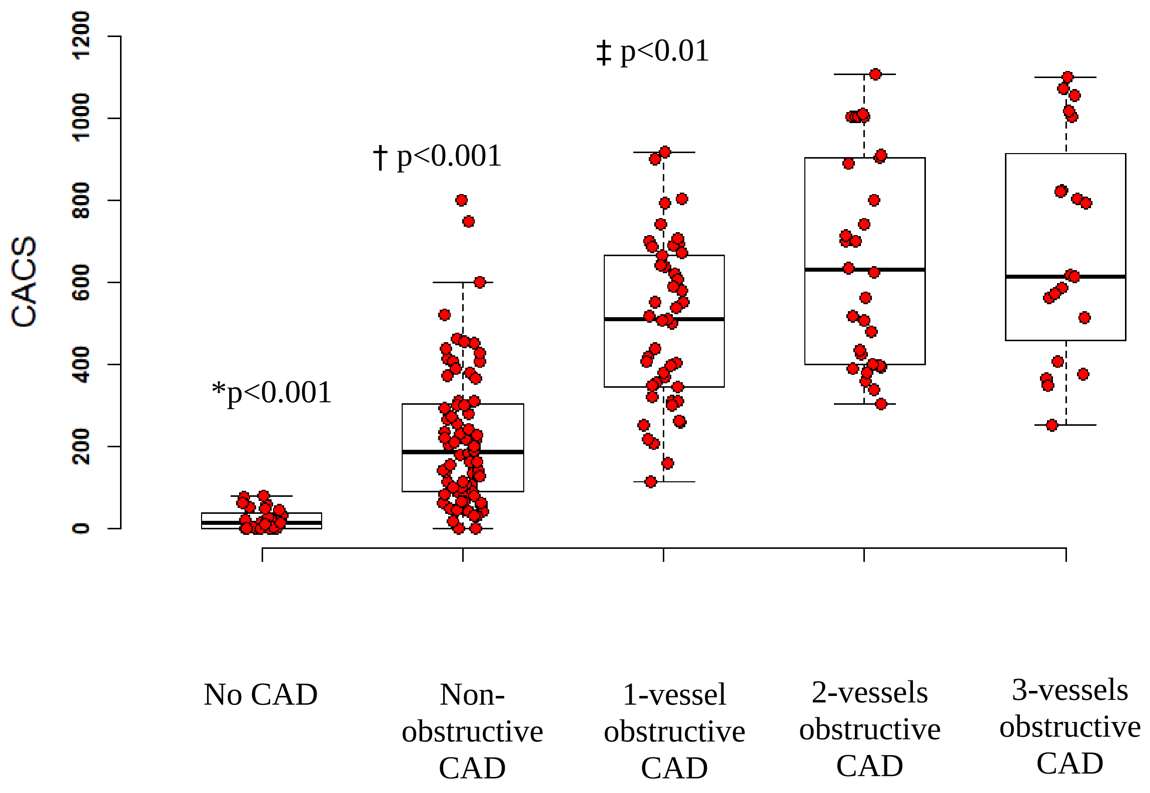

CACS = coronary artery calcium score, CAD = coronary artery disease

*There was a significant difference between the group with no CAD and all other groups

† There was a significant difference between non-obstructive CAD and all other groups

‡ There was a significant difference between 1-vessel obstructive CAD and all other groups

Supplement 2. One-year mortality among 199 consecutive type 2 diabetic patients undergoing partial foot amputation based on coronary computed tomography angiography and coronary artery calcium score

Mortal cases/n (%)	CACS=0 (n=12)	CACS 1-99 (n=38)	CACS 100-399 (n=70)	CACS 400-999 (n=68)	CACS ≥1000 (n=11)
No CAD, n=27	0/10 (0)	0/17 (0)	-	-	-
Non-obstructive CAD, n=77	0/2 (0)	0/21 (0)	4/42 (9.5)	0/12 (0)	-
1-vessel obstructive CAD, n=46	-	-	0/17 (0)	6/29 (20.3)	-
2-vessels obstructive CAD, n=29	-	-	2/7 (25)	4/16 (25)	3/6 (50)
3-vessels obstructive CAD, n=20	-	-	1/4 (25)	10/11 (90.9)	5/5 (100)

CACS=coronary artery calcium score; CAD=coronary artery disease.

Supplement 3. Coronary computed tomographic angiography 18-segments model in type 2 diabetic patients undergoing partial foot amputation.

		No CAD		Non-obstructive CAD				Obstructive CAD					
№	Stenosis	0%		1-30%		31-50%		51-69%		>70%		100%	
		n	%	n	%	n	%	n	%	n	%	n	%
1.	Proximal RCA	140	70.4	13	6.5	28	14.1	11	5.5	7	3.5	-	-
2.	Mid RCA	97	48.7	32	16.1	35	17.6	22	11.1	13	6.5	-	-
3.	Distal RCA	79	39.7	44	22.1	39	19.6	24	12.1	11	5.5	2	1.0
4.	PDA of RCA	157	78.9	25	12.6	10	5.0	1	0.5	6	3	-	-
5.	PLB-RCA	159	79.9	24	12.1	9	4.5	1	0.5	3	1.5	3	1.5
6.	Left main	162	81.4	16	8.0	15	7.5	3	1.5	3	1.5	-	-
7.	Proximal LAD	130	65.3	26	13.1	24	12.1	10	5.0	9	4.5	-	-
8.	Mid LAD	99	49.7	27	13.6	36	18.1	19	9.5	18	9.0	-	-
9.	Distal LAD	96	48.2	37	18.6	31	15.6	7	3.5	26	13.1	2	1.0
10.	Diagonal 1	136	68.7	33	16.7	13	6.6	5	2.5	7	3.5	4	2.0
11.	Diagonal 2	154	77.4	21	10.6	8	4.0	2	1.0	8	4.0	6	3.0
12.	Proximal LCx	145	72.9	23	11.6	20	10.1	5	2.5	6	3.0	-	-
13.	OM1	152	76.4	21	10.6	16	8.0	4	2.0	4	2.0	2	1.0
14.	Mid and distal LCx	120	60.3	30	15.1	24	12.1	10	5.0	14	7.0	1	0.5
15.	OM2	154	78.2	18	9.1	12	6.1	5	2.5	4	2.0	4	2.0
16.	PDA- LCx	159	79.9	16	8.0	15	7.5	3	1.5	5	2.5	1	0.5
17.	Ramus intermedius	166	83.4	17	8.5	13	6.5	1	0.5	2	0.3	-	-
18.	PLVB	176	89.3	11	5.6	6	3.0	3	1.5	1	0.5	-	-

LAD = left anterior descending artery; OM = obtuse marginal; PDA = posterior descending artery; PLB = posterior-lateral branch; PLSA = posterolateral segment artery; PLVB = posterior left ventricular branch; RCA = right coronary artery; RPL = right posterolateral artery.

Abbreviation list and acronyms

ABI, Ankle-brachial index; ANOVA, One-way analysis of variance; BMI, body mass index; CACS, Coronary artery calcium score; CAD, Coronary artery disease; CI, Confidence interval; CCTA, Coronary computed tomographic angiography; CT, Computed tomography; HR, Hazard Ratio; LAD, Left anterior descending artery (LAD); LCx, Left circumflex artery; LEAD, Lower extremity artery disease; MACE, Major adverse cardiovascular events; MI, Myocardial infarction; PA, Physical activity; PAD, Periphery artery disease; RCA, Right coronary artery; PFA, Partial foot amputation; RCRI, Revised cardiac risk index; SD, Standard deviation; T2D, Type 2 diabetes.
